# Kisspeptin enhances brain responses to olfactory and visual cues of attraction in men

**DOI:** 10.1172/jci.insight.133633

**Published:** 2020-02-13

**Authors:** Lisa Yang, Lysia Demetriou, Matthew B. Wall, Edouard G.A. Mills, David Zargaran, Mark Sykes, Julia K. Prague, Ali Abbara, Bryn M. Owen, Paul A. Bassett, Eugenii A. Rabiner, Alexander N. Comninos, Waljit S. Dhillo

**Affiliations:** 1 Section of Endocrinology & Investigative Medicine, Division of Diabetes, Endocrinology and Metabolism, Department of Metabolism, Digestion and Reproduction, Faculty of Medicine, Imperial College London, United Kingdom.; 2 Invicro, Hammersmith Hospital, London, United Kingdom.; 3 Division of Brain Sciences, Faculty of Medicine, Imperial College London, United Kingdom.; 4 Clinical Psychopharmacology Unit, University College London, United Kingdom.; 5 Statsconsultancy Ltd, Bucks, United Kingdom.; 6 Centre for Neuroimaging Sciences, Institute of Psychiatry, Psychology & Neuroscience, King’s College, London, United Kingdom.; 7 Department of Endocrinology, Imperial College Healthcare NHS Trust, London, United Kingdom.

**Keywords:** Endocrinology, Neuroscience, Behavior, Neuroendocrine regulation, Neuroimaging

## Abstract

Successful reproduction is a fundamental physiological process that relies on the integration of sensory cues of attraction with appropriate emotions and behaviors and the reproductive axis. However, the factors responsible for this integration remain largely unexplored. Using functional neuroimaging, hormonal, and psychometric analyses, we demonstrate that the reproductive hormone kisspeptin enhances brain activity in response to olfactory and visual cues of attraction in men. Furthermore, the brain regions enhanced by kisspeptin correspond to areas within the olfactory and limbic systems that govern sexual behavior and perception of beauty as well as overlap with its endogenous expression pattern. Of key functional and behavioral significance, we observed that kisspeptin was most effective in men with lower sexual quality-of-life scores. As such, our results reveal a previously undescribed attraction pathway in humans activated by kisspeptin and identify kisspeptin signaling as a new therapeutic target for related reproductive and psychosexual disorders.

## Abstract 2

Kisspeptin enhances brain processing in response to olfactory and visual cues of attraction and is most effective in men with lower sexual quality of life.

## Introduction

Attraction to another individual is the fundamental initiating step for sexual behavior (1). This innate process relies on the integration of multimodal sensory cues with appropriate emotional and behavioral outputs. However, the intrinsic factors that mediate human attraction remain incompletely understood. Unraveling these integral processes is of major scientific and clinical importance, as related psychosexual disorders affect up to 1 in 3 people worldwide (2, 3), with significant detrimental effects on quality of life, interpersonal relationships, and fertility (4). Furthermore, despite the high clinical burden, our limited understanding of the brain processes governing human psychosexual function has restricted the development of effective treatments (5).

Olfactory and visual signals provide key sensory inputs for sexual attraction. Olfaction is central to many important sexual behaviors, and its role in mate attraction has been extensively studied in nonhuman mammals (6). Olfactory function is also significantly correlated with sexual function in humans (7, 8), and the primary olfactory network has numerous projections to key limbic areas involved in sexual and emotional processing (9). Furthermore, functional neuroimaging has demonstrated that when exposed to a feminine scent, heterosexual men exhibit increased brain activity in limbic regions associated with sexual desire and arousal (10). The visual appreciation of beauty is another fundamental aspect of human attraction, with evidence that prefrontal areas, in particular the medial prefrontal cortex (mPFC), are heavily involved in the human perception of beauty (11–13). Facial beauty provides a symbol of sexual potential, with evidence that men show consistent cross-cultural preferences for certain female facial characteristics (14). Thus, studying factors that may modulate these olfactory and visual cues of attraction can provide important insights into human psychosexual function.

The intrinsic factors that integrate and coordinate these fundamental aspects of human attraction with limbic and reproductive pathways remain as yet unknown. The reproductive hormone kisspeptin is a crucial endogenous activator of the reproductive axis (15–20) and is widely expressed in limbic brain regions along with its cognate receptor (21–24). Furthermore, kisspeptin neurons interconnect olfactory, limbic, and hypothalamic reproductive centers in rodents (25). Studies in animals suggest that kisspeptin influences brain pathways controlling reproductive behaviors (26–28) and is requisite for olfactory system–mediated partner preference (28, 29). Combined, these data led us to postulate that kisspeptin may be an elusive factor in human attraction, which has not been investigated until now to our knowledge. We therefore hypothesized that kisspeptin enhances the brain processing of attraction in humans.

**Figure 1:**
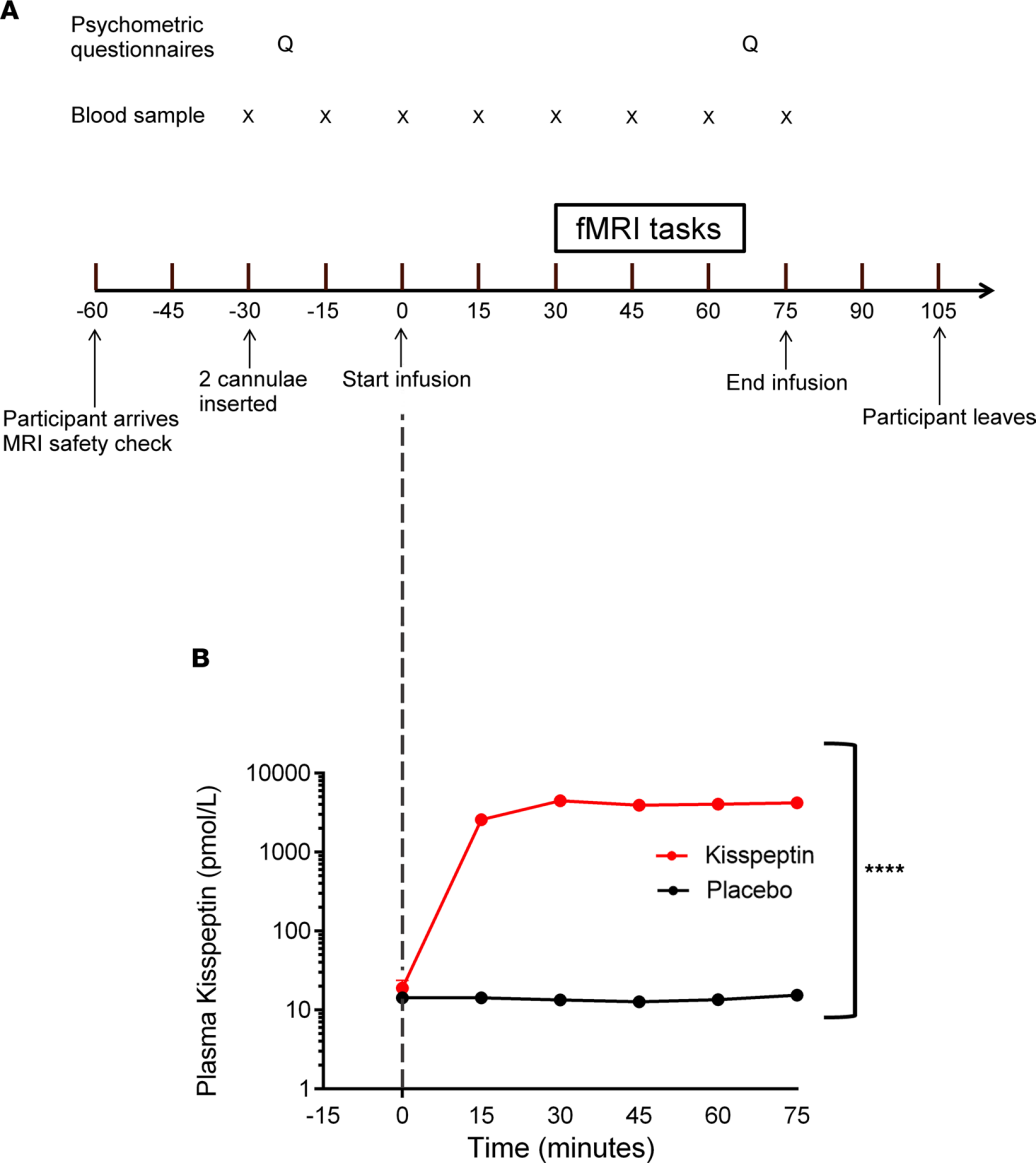
Experimental protocol and effects of kisspeptin administration on circulating kisspeptin levels. (**A**) Thirty-three healthy young men participated in a randomized, double-blind, 2-way crossover, placebo-controlled study. They attended 2 study visits: 1 for intravenous administration of kisspeptin (1 nmol/kg/h) and 1 for intravenous administration of an equivalent volume of placebo (vehicle) for 75 minutes. Blood samples were taken every 15 minutes (X). Participants completed baseline and intrainfusion psychometric questionnaires (Q) and underwent functional MRI (fMRI) scanning while performing olfactory and facial attractiveness tasks. (**B**) Kisspeptin infusion resulted in increased circulating kisspeptin levels (****P < 0.0001, and *n* = 33), reaching a plateau at 30 minutes after initiation, with stable circulating kisspeptin levels during the fMRI and intrainfusion psychometric questionnaires.

To test our hypothesis, we performed a randomized, double-blind, 2-way crossover, placebo-controlled study in 33 healthy, heterosexual men (mean age 24.5 ± 0.7 years, mean BMI 22.9 ± 0.8 kg/m^2^) using functional neuroimaging, hormonal, and psychometric assessments to examine the effects of peripheral kisspeptin (via intravenous infusion) on brain processing during olfactory and facial attractiveness tasks (Figure 1A).

## Results

### Kisspeptin administration increased circulating kisspeptin but not testosterone or cortisol levels.

At baseline (preinfusion), kisspeptin, gonadotropin, and testosterone levels were equivalent between study visits (Supplemental Table 2; supplemental material available online with this article; https://doi.org/10.1172/jci.insight.133633DS1). Subsequently, intravenous kisspeptin infusion (1 nmol/kg/h) significantly increased circulating kisspeptin levels, reaching steady state for the duration of fMRI scanning and psychometric questionnaires (Figure 1B). As expected, kisspeptin administration led to raised luteinizing hormone (LH) levels (Supplemental Figure 1), which indicated that the dose of kisspeptin used was biologically active. We also took precautions to avoid other hormonal confounders. Indeed, kisspeptin administration had no significant effects on testosterone levels during the 75-minute study period because each study was completed before any downstream increases in testosterone (Supplemental Figure 1), which have previously been shown to occur following longer periods of kisspeptin exposure in humans (30). Baseline cortisol levels were also equivalent between study visits, and kisspeptin administration had no significant effects on cortisol levels (Supplemental Figure 1 and Supplemental Table 2).

### Kisspeptin enhanced brain activity in olfactory and limbic circuits governing human sexual behavior, on exposure to a pleasant feminine scent.

**Figure 2:**
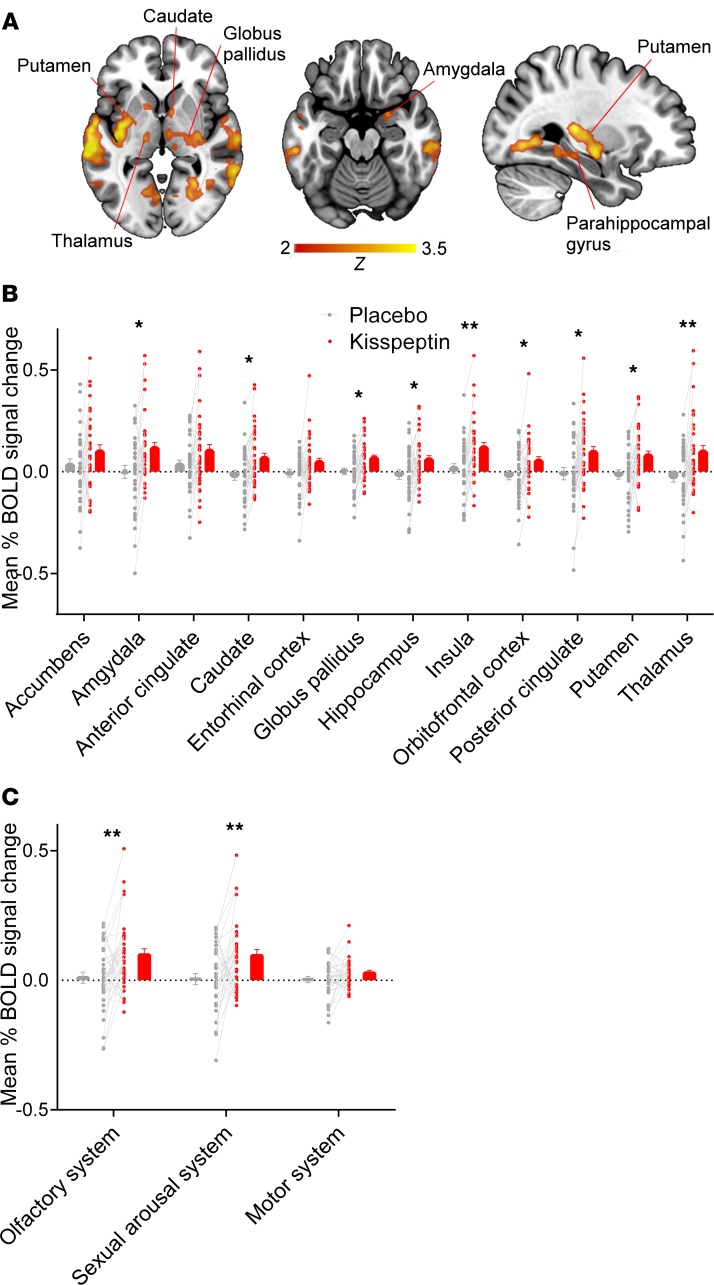
Olfactory task. (**A**) Whole-brain analysis of enhanced blood oxygen level–dependent (BOLD) activity by kisspeptin administration in response to a validated pleasant feminine scent (Chanel No5). Whole-brain voxel-wise analyses with cluster correction (*Z* 2.3, *P* < 0.05, and *n* = 33). (**B**) Kisspeptin enhancement of mean percentage of BOLD signal change in a priori anatomically defined ROI (amygdala: t(32) = 2.743, *P* *=* 0.01; caudate: t(32) = 2.615, *P* = 0.013; globus pallidus: t(32) = 2.566, *P* *=* 0.015; hippocampus: t(32) = 2.235, *P* *=* 0.033; insula: t(32) = 3.105, *P* *=* 0.004; orbitofrontal cortex: t(32) = 2.405, *P* *=* 0.022; posterior cingulate cortex: t(32) = 2.303, *P* *=* 0.028; putamen: t(32) = 2.702, *P* *=* 0.011; thalamus: t(32) = 2.787, *P* *=* 0.009). (**C**) Kisspeptin enhancement of mean percentage of BOLD signal change in functionally defined brain masks during the olfactory task. Olfactory system: t(32) = 2.81, *P* *=* 0.008; sexual arousal system: t(32) = 2.937, *P* *=* 0.006; motor system: t(32) = 1.601, *P* *=* 0.119. Gray indicates placebo; red indicates kisspeptin. Data in graphs (**B** and **C**) depict within-participant paired raw data, mean ± SEM. *P < 0.05, and **P < 0.01, paired 2-tailed *t* test.

During the olfactory task, a feminine scent was delivered nasally, alternating with odorless air as baseline. Chanel No5 was selected as the olfactory stimulus because it has previously been shown to activate limbic regions associated with sexual arousal in a validated fMRI protocol (10). Consistent with this, participants in the current study also validated it as a pleasant and feminine scent (Supplemental Table 1). We observed that kisspeptin significantly enhanced brain activity compared with placebo in key limbic areas related to olfaction and sexual processing (including the amygdala and thalamus), on whole-brain voxel-wise analysis, in response to this pleasant feminine scent (Figure 2A and Supplemental Table 4).

To explore this further, we performed a regions of interest (ROI) analysis (Figure 2B) with a priori–defined regions involved in olfactory and sexual processing and brain regions known to express kisspeptin receptors in humans (9, 21, 31). Kisspeptin significantly increases brain activity in the amygdala, a central component of the primary olfactory cortex (9), as well as the hippocampus, insula, and orbitofrontal cortex (OFC), which form key limbic projections from the primary olfactory cortex and are common substrates for olfactory and emotional processing (9, 31). Furthermore, kisspeptin significantly enhanced activity in the globus pallidus and putamen, which constitute part of the olfactory hedonic processing network, along with the amygdala, hippocampus, and OFC (32). Interestingly, regions associated with reward, motivation, and “romantic love” were also enhanced by kisspeptin, including the thalamus, posterior cingulate cortex (PCC), and caudate (33, 34). Moreover, kisspeptin’s enhancement of thalamus and insula activity corresponded to these established areas of activation during physiological sexual arousal (35). Collectively, these data indicate that kisspeptin augments olfactory as well as sexual and emotional processing in response to pleasurable olfactory stimuli in men.

Next, we used a systems-based approach to investigate the overall effects of kisspeptin on brain systems controlling olfaction and sexual arousal. Using brain masks derived from meta-analytic data (36), we performed a secondary ROI analysis of overall kisspeptin effect compared with placebo on the olfactory system and sexual arousal system, with the motor system as a control. Here, kisspeptin significantly enhanced brain activity in both the olfactory and sexual arousal systems but not in the control motor system, which highlights the specificity of kisspeptin’s effects (Figure 2C).

### Kisspeptin enhanced brain activity in areas governing the evaluation of beauty, on viewing female faces.

**Figure 3:**
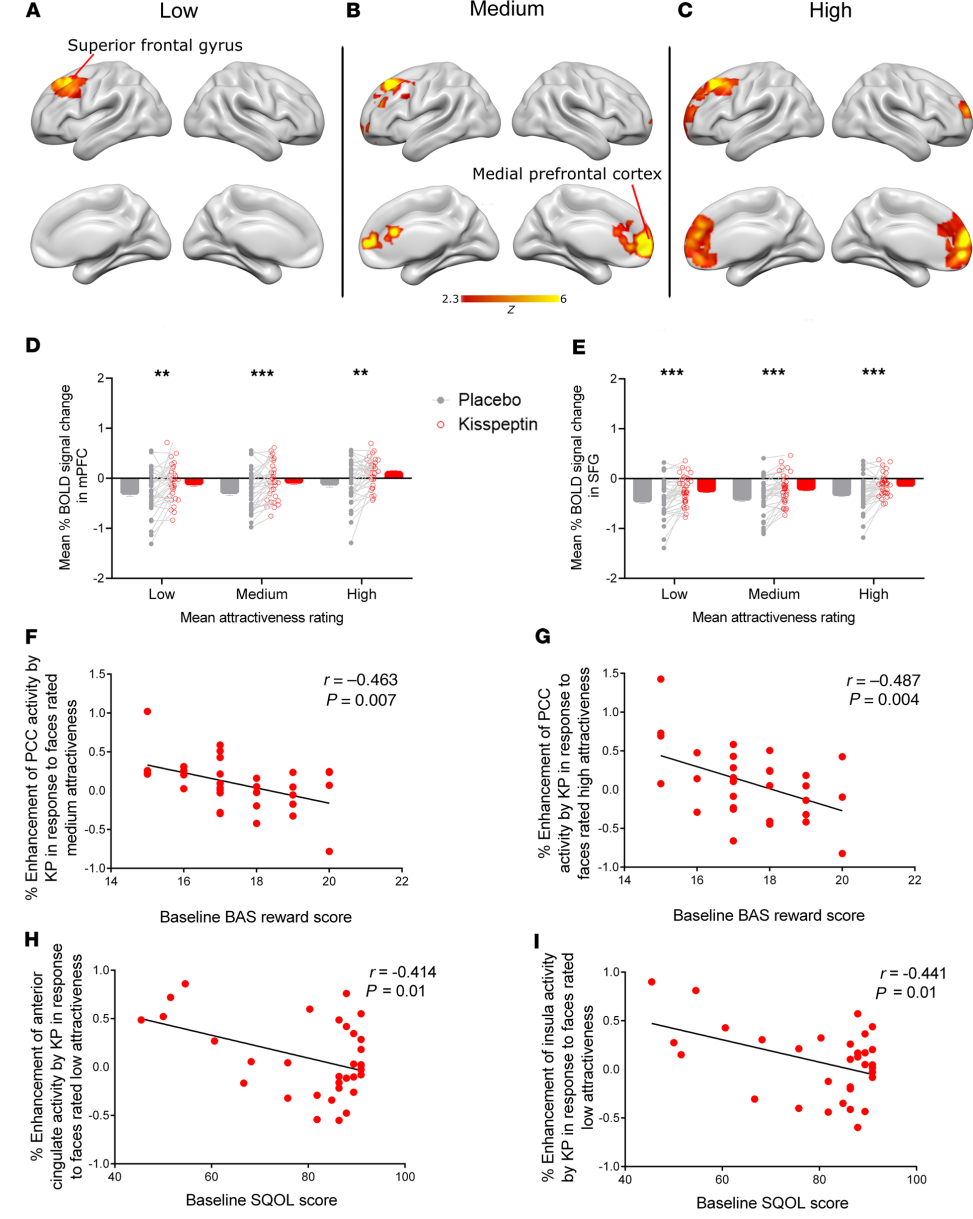
Facial attractiveness task. (**A**–**C**) Whole-brain analysis of kisspeptin-enhanced BOLD activity in response to faces rated (**A**) low, (**B**) medium, and (**C**) high attractiveness (*n* = 33). (**D**) Mean percentage of BOLD signal change in the functionally defined ROI. mPFC, low: t(32) = 2.804, *P* *=* 0.009; medium: t(32) = 4, *P* *<* 0.001; high: t(32) = 3.066, *P* *=* 0.004. (**E**) Mean percentage of BOLD signal change in the functionally defined ROI. SFG, low: t(32) = 3 .966, *P* *<* 0.001; medium: t(32) = 4.567, *P* *<* 0.001; high: t(32) = 3.668, *P* *<* 0.001. (**P < 0.01, and ***P < 0.001, paired 2-tailed *t* test.) (**F** and **G**) Pearson’s correlation between BAS reward score and PCC enhancement by kisspeptin in response to faces rated (**F**) medium (*r* = –0.463, and *P* *=* 0.007) and (**G**) high (*r* = –0.487, and *P* *=* 0.004) attractiveness. (**H** and **I**) Pearson’s correlation between sexual quality-of-life (SQOL) score and enhancement of (**H**) ACC (*r* = –0.414, and *P* = 0.01) and (**I**) insula (*r* = –0.441, and *P* = 0.01) activity by kisspeptin in response to faces of low attractiveness (*n* = 33).

To investigate the effects of kisspeptin on the perception of facial beauty, participants were presented with 60 female faces in random order, selected from a validated database, comprising 3 groups of 20 faces rated in accordance with attractiveness (high, medium, low) by 1087 independent raters (37). Participants viewing faces rated to have high and medium attractiveness exhibited enhanced activity in the mPFC and the superior frontal gyrus (SFG) during kisspeptin administration compared with placebo on whole-brain voxel-wise analyses (Figure 3, B and C, and Supplemental Table 4). Consistent with our data, the mPFC is a well-established area involved in the appreciation of facial beauty (11, 12). In addition, functional neuroimaging has also demonstrated SFG enhancement in response to attractive faces (38), and both the mPFC and SFG express kisspeptin receptors in humans (21, 22). We therefore undertook ROI analyses of both regions to explore this further. In keeping with the literature, graded responses in overall mPFC and SFG activity were observed with increasing facial attractiveness in the placebo groups (Figure 3, D and E) (12, 38). Remarkably, kisspeptin significantly enhanced both mPFC and SFG activity compared with placebo across all 3 categories of attractiveness (Figure 3, D and E). Together, these findings demonstrate that kisspeptin augments the processing of facial beauty across a spectrum of facial attractiveness, therefore serving as an amplifier within the human neural aesthetic circuitry involved in the assessment of facial beauty.

We also explored areas known to express kisspeptin receptors and limbic regions involved in sexual arousal by performing an ROI analysis based on a priori–defined brain regions (accumbens, amygdala, anterior cingulate cortex, caudate, globus pallidus, hippocampus, PCC, putamen, and thalamus) (21, 39). Our results revealed that kisspeptin’s enhancement of brain activity on viewing a female face was specific to the mPFC and SFG (established aesthetic regions) because kisspeptin did not significantly modulate brain activity in these other regions (Supplemental Figure 2).

### The effects of kisspeptin on brain activity were more pronounced in men with lower baseline reward and sexual quality of life.

To assess behavioral and functional relevance for our brain activity data, participants also completed standardized, validated psychometric questionnaires (Supplemental Tables 1 and 2), including questionnaires designed to assess reward and sexual quality of life (40–43). Correlation analyses between these behavioral parameters and neuroimaging data demonstrated that participants with lower baseline behavioral activation system (BAS) reward scores (40) showed greater kisspeptin-enhanced brain activity in the PCC, on viewing faces rated high (*r* = –0.487, and *P* = 0.004, Figure 3G) and medium attractiveness (*r* = –0.463, and *P* = 0.007, Figure 3F). Similarly, participants who reported a lower baseline sexual quality of life score (43) showed much greater kisspeptin-enhanced activity in the anterior cingulate cortex (*r* = –0.414, and *P* = 0.01) and insula (*r* = –0.441, and *P* = 0.01) on viewing faces with low attractiveness (Figure 3, H and I), key regions involved in reward and incentive motivation (44, 45). These behavioral findings therefore provide crucial relevance for our brain activity data and lay the foundation for future clinical applications of kisspeptin.

## Discussion

In this study, we demonstrate for the first time to our knowledge that the reproductive hormone kisspeptin enhances brain activity specifically in response to olfactory and visual cues of attraction in healthy men. During the olfactory task, areas significantly enhanced by kisspeptin included key limbic regions known to be involved in human olfactory processing and sexual arousal, but crucially, kisspeptin did not affect the motor system, which was used as a control. Similarly, during the facial attractiveness task, kisspeptin selectively amplified the mPFC and SFG (established aesthetic regions) (Figure 3, A–C). In contrast, other limbic regions were unaffected by kisspeptin during this facial attractiveness task (Supplemental Figure 2), unlike in the olfactory task (Figure 2B). These data highlight our findings of targeted region-specific effects of kisspeptin, dependent on the nature of the attraction cue (olfactory or visual).

Studies in different animal species have shown varying anatomical expression patterns for kisspeptin and its receptor, which may be due to species and methodological differences (23, 24). Therefore, to select brain regions of interest for our fMRI analyses, we used the available data describing distribution of human kisspeptin receptor mRNA (21, 22). Our results show that peripheral kisspeptin administration enhanced brain regions matching areas where human kisspeptin receptor is expressed, suggesting a direct receptor-mediated action of kisspeptin in these brain regions. Given these findings, it is important to consider how peripherally administered kisspeptin can reach brain regions of interest. Gonadotropin-releasing hormone (GnRH) neurons extend dendritic terminals beyond the blood-brain barrier; thus, peripheral kisspeptin is capable of stimulating GnRH, and in turn, LH, without needing to cross the blood-brain barrier (19). However, different isoforms of kisspeptin have been shown to have varying degrees of blood-brain barrier penetrance. Peripherally administered kisspeptin-54, as used in this study, is capable of reaching both GnRH cell bodies beyond the blood-brain barrier (46) as well as numerous limbic brain structures known to express kisspeptin and its receptor (39).

It is also important to consider additional pathways that may be involved in exerting the observed downstream effects of kisspeptin on the brain. Kisspeptin activates GnRH neurons, and these have been identified in several areas of the brain in humans, including the cerebellum, thalamus, anterior olfactory areas, amygdala, stria terminalis, ventral pallidum, and putamen (47–49). Interestingly, we identified enhanced brain activity due to kisspeptin administration in several areas that are not known to contain GnRH neurons or GnRH receptors in humans (including the caudate, globus pallidus, insula, and PCC) (47–50), which suggests there are GnRH-independent actions of kisspeptin in these regions. Furthermore, there is recent functional evidence that certain kisspeptin-induced sexual behaviors can occur independent of GnRH. These include kisspeptin-induced lumbar lordosis in mice (28) and kisspeptin-stimulated erections in rats (26). In addition, data from animal studies demonstrate that kisspeptin also interacts with numerous other neuropeptide systems, including serotonin (51, 52), dopamine (25), vasopressin (25), GABA (53), glutamate (54), and nitric oxide (16, 28, 55). Thus, the fMRI changes that we have shown may be the product of direct kisspeptin effects on its receptor as well as interactions between kisspeptin and these other downstream neural systems.

In this study, we have shown that kisspeptin administration can enhance brain processing in olfactory and sexual arousal systems of healthy men in response to a validated olfactory cue. In animal studies, opposite-sex odors or pheromones have been used to elicit olfactory system–driven behavioral and endocrine responses (28, 56). In humans, the evidence for behavioral and physiological responses to opposite-sex odors is conflicting (57); therefore, we selected Chanel No5 as a widely recognized and validated feminine scent (10) to activate brain areas responding to a consciously perceived female olfactory cue so that kisspeptin’s effects on these regions could be robustly assessed. Our results show that kisspeptin administration enhances brain responses to a feminine scent in several key components of the limbic system that are involved in olfactory processing, hedonic valuation of olfactory stimuli, and sexual arousal networks (9, 31). Intriguingly, the same enhancement in limbic activity is not seen during the facial attractiveness task. Instead, kisspeptin significantly enhanced activity in the mPFC and SFG, 2 prefrontal regions that are known to govern the perception of facial beauty (11, 12, 38). This is in keeping with previous data indicating that limbic reward pathways are not necessarily involved in facial aesthetic assessment (11). Thus, our data demonstrate that kisspeptin’s effects on brain activity are specific to the relevant regions involved in different sensory modalities of attraction (olfactory or visual).

Furthermore, we observed significant correlations between kisspeptin-enhanced brain activity and important psychometric parameters, thereby providing key behavioral and functional relevance for the observed brain changes. Indeed, greater kisspeptin enhancement was observed in the PCC on viewing attractive faces in men with lower baseline reward scores. The PCC is implicated in romantic love (34), and its activity is known to vary with emotional memory and reward (58). Thus, kisspeptin may act to enhance emotional salience and reward processing in the PCC on viewing attractive faces in order to rebalance a lower reward drive in these individuals, in favor of promoting sexual attraction. A similar and perhaps even more intriguing relationship was demonstrated between kisspeptin’s enhancement of ACC and insula activity in response to female faces and low sexual quality of life. The putamen is a dopamine-rich area that responds to visual sexual stimuli (59), and both the ACC and insula are areas implicated in sexual arousal (60), facial attraction (12, 38), and motivation toward reward (44, 45). Thus kisspeptin’s enhancement of these brain regions on viewing attractive faces may serve to strengthen feelings of reward, attraction, and incentive motivation in individuals experiencing lower sexual quality of life, ultimately to encourage reproduction at a population level. Collectively, these findings provide key behavioral and functional relevance for kisspeptin’s enhancement of brain activity on viewing attractive faces and lay the foundation for potential clinical applications of our data for patients with common reproductive and psychosexual disorders.

We demonstrate these effects on human brain processing through the administration of kisspeptin, achieving plasma kisspeptin levels similar to the levels required to restore physiological LH pulsatility in women with hypothalamic amenorrhoea (61). Equivalent plasma concentrations of kisspeptin are also observed physiologically in normal pregnancy (62). Although kisspeptin can modulate GnRH pulsatility in humans (63–65), current evidence suggests that its effects on behavior do not rely on changes in GnRH pulsatility because LH remained elevated and nonpulsatile in this as well as previous work showing kisspeptin-induced behavioral changes (39). In addition and as discussed earlier, kisspeptin exerts certain GnRH-independent behavioral effects, and we have previously seen a reduction in negative mood and sexual aversion without any observed modulations of LH pulsatility (39). Taken together, these data suggest that the behavioral effects of kisspeptin can be achieved without modulation of downstream GnRH pulsatility. As such, this introduces a new and exciting avenue for kisspeptin therapeutics in the management of psychosexual disorders in addition to common reproductive disorders, with further studies necessary to fully elucidate the role of endogenous kisspeptin activity.

When considering attraction, it is also important to bear in mind that although visual and olfactory inputs are fundamental cues of attraction in many species (1), human sexual responses are complex and influenced by multiple additional components, such as context, personality traits, and body language (66), which may affect individual perceptions of attractiveness. In our study, we controlled for some of these factors by omitting motion picture and audio stimuli as potential confounders in participant responses; however, it would be interesting to study these other components of attraction in the future. In addition, we ensured that only investigators of the same sex conducted physical interactions with participants during the study (e.g., administering psychometric questionnaires, cannulation, and blood sampling) to standardize any effects of experimenter sex (67). Importantly, we also took precautions to measure related hormones that could confound our results. Indeed, we observed that kisspeptin had no effect on cortisol or testosterone levels during this time course (Supplemental Figure 1).

In summary, our findings reveal a previously undescribed kisspeptin-activated attraction pathway, uniting 2 major sensory inputs of human attraction with corresponding neural processing and reproductive hormonal control. Furthermore, kisspeptin’s enhancement of key brain areas within these pathways is augmented in men with lower behavioral reward drive and sexual quality of life, providing crucial functional relevance to our imaging findings. Collectively, this has important implications for our understanding of human psychosexual function and its endocrine control. Crucially, our data also lay the foundation for manipulation of these newly identified kisspeptin-mediated pathways to deliver much-needed clinical strategies for individuals suffering from related common reproductive and psychosexual disorders.

## Methods

### Participants.

Previous work demonstrates that kisspeptin enhances task-based brain activity, measured by percentage of BOLD signal change, in the amygdala by mean 0.74% and standard deviation 0.38% compared with placebo (mean 0.48%, standard deviation 0.51%) (39), and we expected a similar response in this study. Using these data, with α 0.05, with power 0.8, and assuming correlation between means of 0.40, a power calculation was performed, resulting in a sample size of 31. This is in line with previous fMRI studies and empirically derived estimates of optimal sample sizes in fMRI studies (39, 68, 69). To allow for natural variation in responses, dropouts, and exclusions, 36 participants were recruited via advertisements. One participant withdrew, 1 participant did not complete the fMRI tasks, and 1 participant was excluded due to a change in health status during the study, giving a final study group of 33 healthy young men. To confirm eligibility, participants attended a medical screening appointment. Participants were free of current and past physical or psychiatric illness and were naive to psychoactive substances, prescribed or illicit. Heterosexuality was determined by a Kinsey score of 0 (70), and normal olfactory function was assessed by the Brief Smell Identification Test (71). All participants had normal basal reproductive hormone levels (Supplemental Table 1). Specific screening was also undertaken to exclude participants with any history of sexual aggression/abuse/phobia or psychotherapy/counseling. In addition, tobacco smokers were excluded because smoking is associated with olfactory deficits that could interfere with the olfactory fMRI task (72). All participants were right-handed and had normal or corrected-to-normal vision.

### Study design.

The participants completed 2 MRI study visits each, as part of a randomized, double-blind, 2-way crossover, placebo-controlled protocol. Therefore, this was a within-participant design study, in which the participants acted as their own controls, thereby minimizing variability and enhancing power. All the studies commenced in the morning to control for time-dependent hormonal changes. Participants were asked to abstain from sexual activities from midnight before the study visit because prior sexual activity can affect testosterone levels (73, 74). In addition, participants were asked to abstain from alcohol and caffeine for the same period of time and to consume a normal breakfast on the morning of their study visit.

On arrival, participants were asked to change into loose hospital scrubs and relax in a supine position for 30 minutes. Intravenous cannulae were then inserted into each antecubital fossa for infusion of kisspeptin or placebo and blood collection (at time points –30, –15, 0, 15, 30, 45, 60, and 75 minutes) (Figure 1A). Participants also completed psychometric questionnaires as detailed below. At time point 0 an infusion of kisspeptin or placebo was commenced lasting 75 minutes. The fMRI tasks were initiated at 30 minutes from the start of infusion to allow plasma kisspeptin levels to reach steady state (Figure 1B).

### Assays.

Blood was collected to measure circulating kisspeptin, LH, follicle stimulating hormone, and testosterone levels, as previously described (30), and to confirm that baseline reproductive hormone levels were equivalent between study visits (Supplemental Table 2). Cortisol was measured on serum samples using an automated delayed 1-step immunoassay (Abbott Diagnostics) with chemiluminescent microparticle immunoassay technology. The precision of the assay was 10% or less total coefficient of variation for serum samples, with values between 83 nmol/L and 966 nmol/L. The functional sensitivity of the assay was 28 nmol/L or less, and the limit of detection was 22 nmol/L or less.

### Behavioral assessments.

Participants were asked to complete a set of psychometric questionnaires before their first MRI scan (Supplemental Table 1). The Patient Health Questionnaire-9 was used to screen for depression, and those scoring above the threshold for depressive disorder were excluded from the study (75). The State-Trait Anxiety Inventory, form Y, questionnaire (76) excluded anxiety traits in our cohort as all scores were within normal range (Supplemental Table 1). The Behavioral Inhibition and Activation System Scales were used to assess sensitivity to anticipation of punishment and to reward (40). Subjective perceptions of happiness and general satisfaction with life were measured using the Subjective Happiness Scale (77) and the Satisfaction with Life Scale (78), respectively. Additionally, baseline sexual quality of life was assessed using the Sexual Quality of Life Questionnaire (43). The Sexual Desire Inventory 2 formally assessed frequency and intensity of desire in normative circumstances of both dyadic (with partner) and solitary sexual desire (42). The International Index of Erectile Function was used to screen for the 5 domains of male sexuality (desire, erectile function, intercourse satisfaction, orgasmic function, and overall satisfaction) (79), with normal baseline results in all participants (Supplemental Table 1). A second set of questionnaires was also completed by participants before and during their infusions (kisspeptin or placebo) to assess for sexual desire and emotional state in the present moment, with no differences observed between kisspeptin and placebo visits (Supplemental Tables 2 and 3). These included the Sexual Arousal and Desire Inventory to evaluate physiological, cognitive-emotional, and aversive or inhibitory components within the subjective experience of sexual desire and arousal (80) and the Profile of Mood States short form for adults, a 37-item questionnaire validated for the assessment of psychological distress using the domains of fatigue, vigor, anxiety, anger, depression, confusion, and friendliness (81).

### fMRI procedure.

During the MRI session, a series of anatomical and functional brain scans were performed. During the functional tasks, a mirror mounted on the head coil was used to view a screen at the rear of the scanner bore, onto which the stimuli were projected. To respond to the tasks, the participants were equipped with a custom-made, 5-button, MRI-compatible response box. In addition, a pulse oximeter and a respiratory belt were used to monitor physiological data by means of a standard data-recording system (AD Instruments PowerLab) in the control room. Kisspeptin and placebo infusions were administered via a Medrad Spectris Solaris MRI-compatible injection system controlled from a remote panel in the control room.

### Olfactory task.

A block design with 20 blocks was used for this task, whereby the participants received nasal delivery of Chanel No5 (concentration as per manufacturer) for 6 seconds followed by 20 seconds of odorless air as baseline. To keep the participants alert, a star appeared on the screen at random times during the task, and the participants were instructed to respond by pressing a button with their index finger on the response box. An ETT1 6-channel olfactometer (Emerging Tech Trans) was used to deliver the scent during the olfactory task. The olfactometer was connected to an odorant carrier in the MRI suite, which transported the scent to the participants via a length of Teflon tubing connected to nasal prongs worn by the participants, which allowed the olfactory stimulus to be delivered at a constant rate to the nasal passage. All participants identified Chanel No5 as pleasant and feminine at baseline (Supplemental Table 1). Participants were also asked to rate the scent during each study visit, and there were no differences in ratings between kisspeptin and placebo visits.

### Facial attractiveness task.

To investigate kisspeptin’s effects on brain responses to varying levels of facial attractiveness on viewing opposite-sex faces, participants were presented with images of female faces from the validated Chicago Faces Database (37). Sixty faces were selected comprising of 3 groups of 20 faces rated in accordance with attractiveness (high, medium, low) by 1087 independent raters (37). Changes in brain activity in response to viewing faces of high, medium, and low attractiveness were compared between kisspeptin and placebo visits, with each participant acting as his own control. An event-related design was used in which each face was presented once for 4 seconds, followed by a jittered intertrial interval of 2 to 10 seconds. To ensure alertness, the participants were asked to rate the attractiveness of each face on a 5-point scale ranging from “very unattractive” to “very attractive” using the 5-button response box. The participants’ ratings were in agreement with the independent raters, and no differences were observed between kisspeptin and placebo visits.

### MRI acquisition.

Imaging data were acquired using a 3T Siemens Trio scanner with a 32-channel phased-array head coil. The anatomical images were acquired at the beginning of each scan using a T1-weighted MPRAGE pulse sequence (1-mm isotropic voxels, repetition time [TR] = 2300 ms, echo time [TE] = 2.98 ms, flip angle = 9°). For the acquisition of functional images, a multiband sequence with acceleration factor 2 was used with the following parameters for the facial attractiveness task: 3-mm voxels, TR = 1 s, TE = 30 ms, flip angle = 80°, 36 axial slices; and for the olfactory task: 2-mm voxels, TR = 1.5 s, TE = 30 ms, flip angle = 80°, 54 axial slices.

### fMRI data analysis.

Imaging analysis was performed using FSL. Pre-processing included motion correction, smoothing (6 mm), registration to a standard template (MNI152), and high-pass filtering (0.01 Hz). A general linear model analysis modeled the occurrence of the stimuli and included their temporal derivatives and head motion regressors as confounds. Group analyses were random effects (FLAME-1) models, with statistical maps thresholded at *Z* = 2.3, and *P* < 0.05 (cluster corrected). Group-mean analyses including both kisspeptin and placebo visits for both tasks showed that the tasks worked effectively (Supplemental Figures 3 and 4). Group-mean analysis for the scent trials against baseline in the olfactory task showed increased activity in key parts of the human olfactory pathway (Supplemental Figure 3). Similarly, a group-mean analysis for the facial attractiveness task showed strong activation in the visual cortex, the frontal lobe, and striatal areas in the 3 attractiveness groups (high, medium, low) (Supplemental Figure 4).

A set of a priori–selected brain regions defined in standard stereotactic space using the Harvard-Oxford atlases (https://fsl.fmrib.ox.ac.uk/fsl/fslwiki/) were used to extract data for ROI analyses. The ROI were selected based on evidence showing presence of kisspeptin receptors in specific areas of the human limbic brain (21) and brain regions that are crucial parts of the olfactory pathway (9). This set consisted of the following regions: accumbens, amygdala, anterior cingulate cortex, caudate, entorhinal cortex, globus pallidus, hippocampus, insula, OFC, PCC, putamen, and thalamus. Subsequently, to explore kisspeptin’s effect on olfaction and reproduction further, we performed ROI analysis using olfactory and sexual arousal brain masks derived from meta-analytic data within the Neurosynth database (http://neurosynth.org/). A motor cortex mask was used as a control. For the facial attractiveness task, the mPFC and SFG masks were functionally defined based on the group-mean results across all participants and both conditions (kisspeptin and placebo). In addition, an ROI analysis was performed (Supplemental Figure 2) based on a priori–defined brain regions comprising areas known to express kisspeptin receptors and areas involved in sexual arousal (accumbens, amygdala, anterior cingulate cortex, caudate, globus pallidus, hippocampus, PCC, putamen, and thalamus) (21, 39).

A repeated-measures, 2 (treatment kisspeptin/placebo) by 14 (ROI) ANOVA was performed for the ROI analysis of the olfactory task. The results showed a significant effect for the ROI and treatment main effects (*P* < 0.01) but no significance in the interaction between the two. For the facial attractiveness task, a repeated-measures, 2 by 3 by 11 (ROI) ANOVA was used to assess effects between ROI, attractiveness, and treatment (kisspeptin/placebo). The results showed significant effects for the 3-way interaction (*P* < 0.01), the 2-way interaction between ROI and attractiveness (*P* < 0.01), and ROI and attractiveness as main effects (*P* < 0.01).

### Statistics.

Statistical analyses were performed in collaboration with a statistician. Data were normally distributed by Kolmogorov testing. Individual paired 2-tailed *t* tests were performed to investigate the kisspeptin effect on each individual region of interest. Pearson’s correlation was used to assess correlations between brain activity and psychometric measures, with data adjusted for visit order where necessary. An α threshold of *P* < 0.05 identified statistical significance in the ROI analyses, but a reduced threshold of *P* < 0.01 was used in the correlation analysis to adjust for the number of analyses performed, in line with previous work (39). Differences between baseline and change in psychometric scores during kisspeptin compared with placebo visits were assessed using multilevel linear regression corrected for visit order.

### Study approval.

The study was performed in accordance with the Declaration of Helsinki. All participants gave written informed consent before inclusion in the study. The study was approved by the regional ethics committee (Riverside Research Ethics Committee, London, United Kingdom, REC 17/LO/1504).

## Author contributions

LY, LD, MBW, EAR, ANC, and WSD conceived the study and designed the protocol. LY, LD, EGAM, DZ, MS, and JKP collected the data. LY, LD, MBW, AA, PAB, and ANC analyzed the data. LY, LD, MBW, BMO, ANC, and WSD prepared the manuscript. ANC and WSD supervised all aspects of the work.

## Supplementary Material

Supplemental data

## Version 1 02/13/2020

Electronic publication
