# Supplementary material for: Kisspeptin enhances brain responses to olfactory and visual cues of attraction in men
Source: JCI Insight. 2020 Feb 13;5(3):e133633. doi: 10.1172/jci.insight.133633 (PMC7098781; doi:10.1172/jci.insight.133633)
Supplement: Supplemental data [file jciinsight-5-133633-s197.pdf]

Kisspeptin Enhances Brain Responses to Olfactory and Visual Cues of Attraction in Men

Authors: Lisa Yang[1#], Lysia Demetriou[1,2#], Matthew B Wall[2,3,4], Edouard GA Mills[1], David Zargaran[1], Mark Sykes[1], Julia K Prague[1], Ali Abbara[1], Bryn M Owen[1], Paul A Bassett[5], Eugenii A Rabiner[2,6], Alexander N Comninos[1,7]*, Waljit S Dhillo[1]*.

# Joint first authors

Affiliations:

[1] Section of Endocrinology & Investigative Medicine, Imperial College London, UK. [2] Invicro, Hammersmith Hospital, London, UK. [3] Division of Brain Sciences, Imperial College London, UK. [4] Clinical Psychopharmacology Unit, University College London, UK. [5] Statsconsultancy Ltd, Bucks., UK. [6] Centre for Neuroimaging Sciences, Institute of Psychiatry, Psychology and Neuroscience, King's College, London, UK. [7] Department of Endocrinology, Imperial College Healthcare NHS Trust, London, UK.

Corresponding authors:

Dr Alexander N. Comninos: a.comninos@imperial.ac.uk

& Professor Waljit S. Dhillo: w.dhillo@imperial.ac.uk

Department of Investigative Medicine,

Imperial College London,

6th Floor, Commonwealth Building,

Hammersmith Hospital Campus, Du Cane Road,

London, W12 ONN, UK.

Tel: +44 208 383 3242.

Conflict of Interest Statement: The authors have declared that no conflict of interest exists.

Publishing under Creative Commons CC-BY license required.

Supplementary Figures and Tables

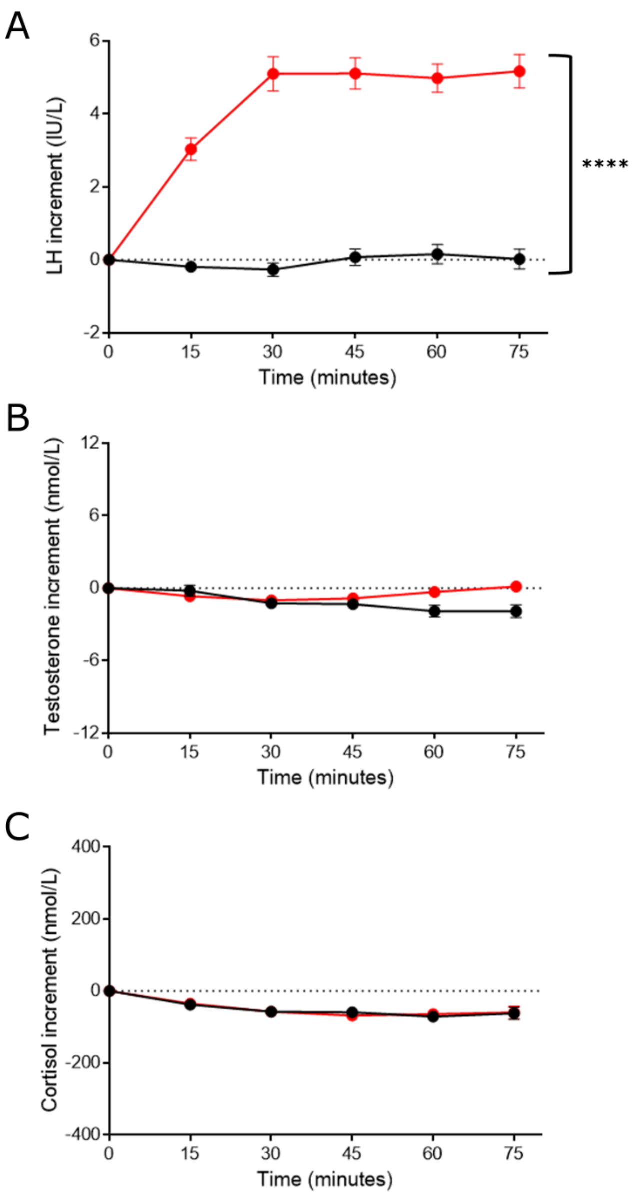

Supplemental Figure S1. Effects of kisspeptin administration on hormone levels.

(A) Kisspeptin increased circulating LH levels. Kisspeptin had no effect on circulating **(B)** testosterone and **(C)** cortisol levels in the blood. Data depict mean±SEM. *****P*<0.0001, two-way ANOVA, *n*=33.

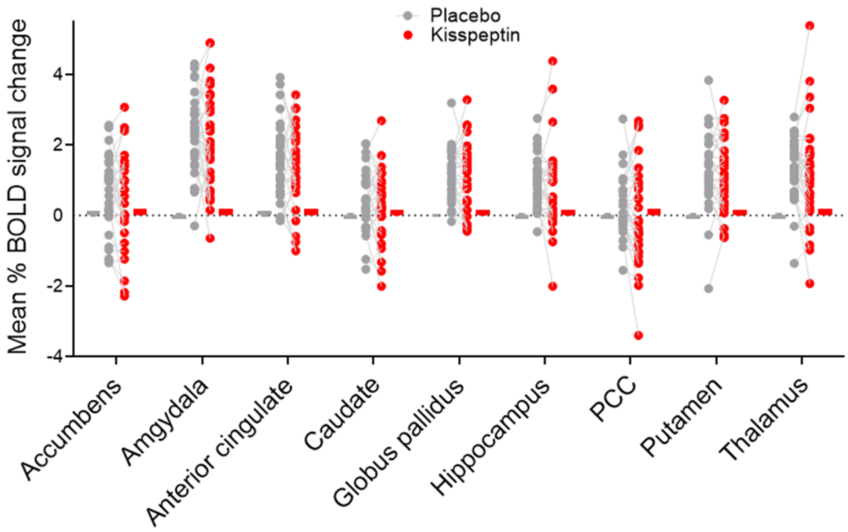

Supplemental Figure S2. Mean % BOLD signal change in *a priori* anatomically defined ROIs during facial attractiveness task.

ROI analysis based on *a priori* defined brain regions comprising areas known to express kisspeptin receptors and areas involved in sexual arousal showed no significant effect of kisspeptin across all face conditions. Data in graph depict within participant raw data, mean±SEM. *n*=33.

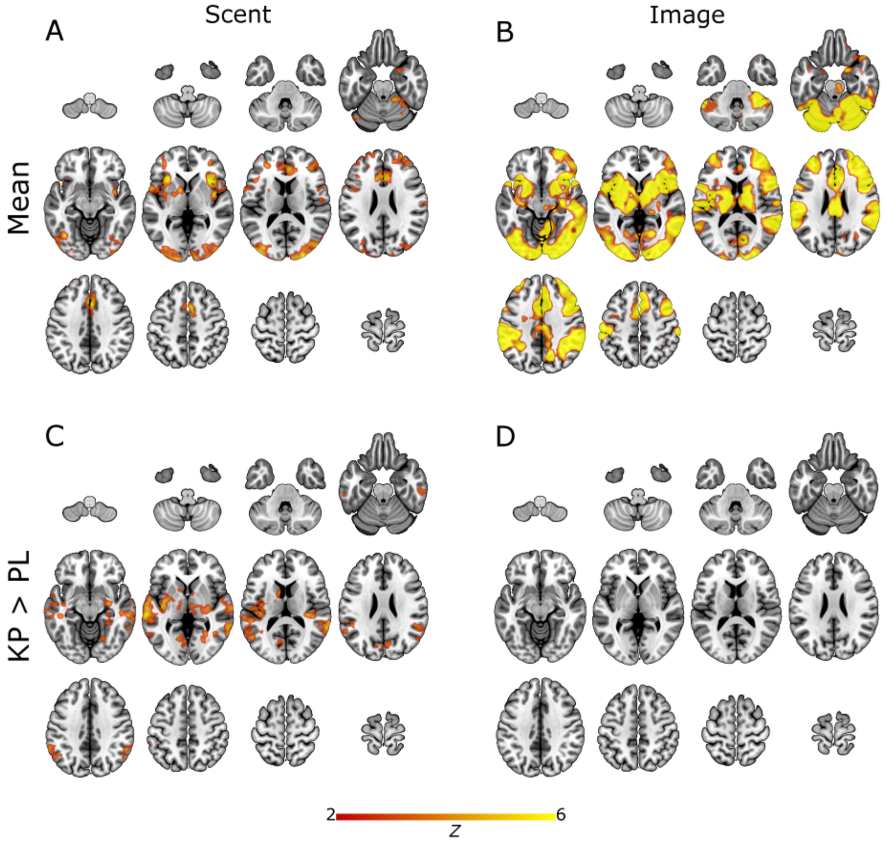

Supplemental Figure S3. Task effects and kisspeptin modulation of these effects in response to the olfactory task. (A-B) Analyses of the main effects of stimulus type (all subjects, both treatments averaged) for **(A)** scent and **(B)** image trials. **(C-D)** Within-subject analyses of the effect of kisspeptin (KP) vs. placebo (PL) for **(C)** scent and **(D)** image trials. Positive voxel values represent an increase in activity during kisspeptin infusion. All statistical maps thresholded at Z=2.3, *P*<0.05 (cluster corrected for multiple comparisons), *n*=33.

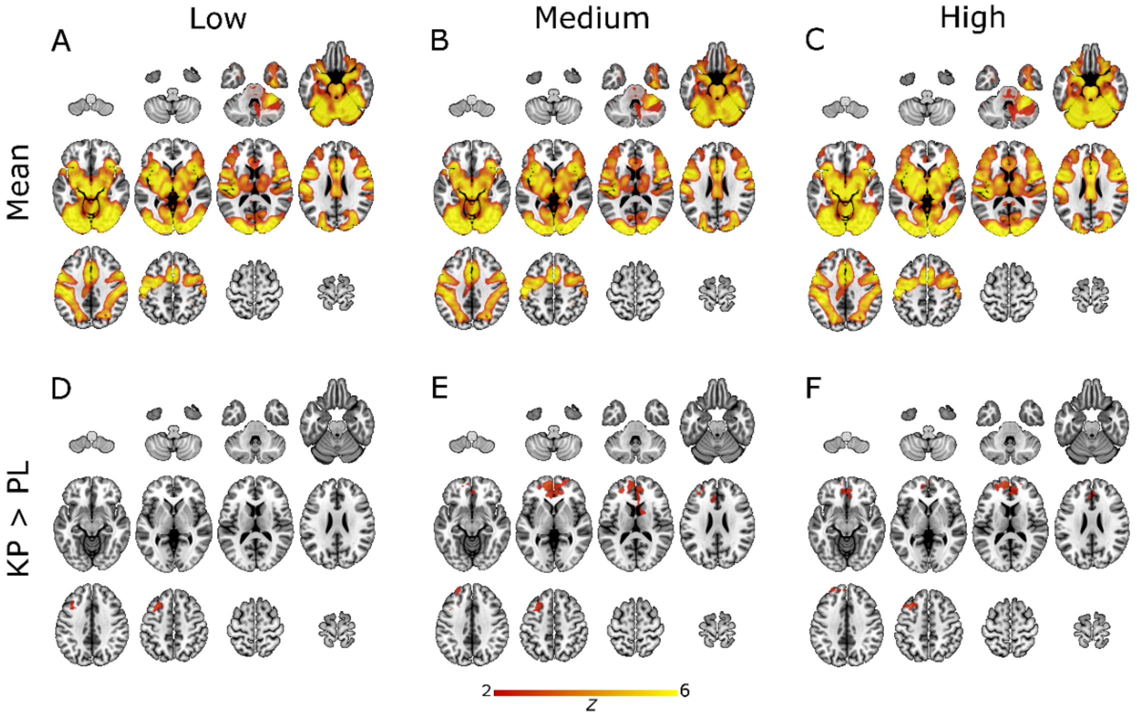

Supplemental Figure S4. Task effects and kisspeptin modulation of these effects in response to facial attractiveness.

(A-C) Analyses of the main effects of stimulus type (all subjects, both treatments averaged) for low, medium and high attractiveness faces (A-C, respectively). **(D-F)** Within-subject analyses of the effect of kisspeptin (KP) vs. placebo (PL) for low, medium and high attractiveness faces. Positive voxel values represent an increase in activity during kisspeptin infusion. All statistical maps thresholded at Z=2.3, *P*<0.05 (cluster corrected for multiple comparisons), *n*=33.

Supplemental Table S1: Participant clinical and psychometric characteristics.

		**Healthy Men (*n* = 33)**
		Mean±SEM
**Age (years)**		24.6 ± 0.7
**BMI (kg/m^2)**		22.9 ± 0.3
**Baseline Reproductive Hormones**		
	Kisspeptin (pmol/L)	17.9 ± 2.4
	LH (IU/L)	2.9 ± 0.2
	FSH (IU/L)	2.9 ± 0.3
	Testosterone (nmol/L)	20.8 ± 1.0
	Cortisol	302.5 ± 13.3
**Number of sexual partners in the last year**		1.8 ± 0.3
**Frequency of sexual intercourse per month**		5.6 ± 0.9
**Hours viewing pornographic material per week**		1.1 ± 0.2
**PHQ-9**		1.2 ± 0.2
**BIS**		19.4 ± 0.6
**BAS**		
	Drive	11.8 ± 0.3
	Fun	12.4 ± 0.4
	Reward	17.5 ± 0.3
**IIEF**		
	Erectile Function	26.8 ± 1.0
	Orgasmic Function	8.7 ± 0.4
	Sexual Desire	7.8 ± 0.2
	Intercourse Satisfaction	10.5 ± 0.8
	Overall Score	8.4 ± 0.3
**STAI-Y Trait**		37.1 ± 1.3
**SDI (Sexual Desire Inventory)**		
	Dyadic	47.2 ± 1.5
	Solitary	17.4 ± 0.9
	Total	68.4 ± 2.2
**SQOL**		80.4 ± 2.4
**SwLS**		24.8 ± 0.8
**SHS**		17.6 ± 0.4
**Baseline Scent Ratings**		
	Pleasant	3.4 ± 0.1
	Feminine	4.7 ± 0.2

BMI = Body Mass Index; LH = Luteinizing Hormone; FSH = Follicle Stimulating Hormone; PHQ-9 = Patient Health Questionnaire-9 to screen for depression; BIS = Behavioral Inhibition System Scale to assess sensitivity to anticipation of punishment; BAS = Behavioral Activation System Scale to assess sensitivity to desired goals, fun and reward; IIEF = International Index of Erectile Function to screen for normal male sexual function; STAI-Y Trait = State-Trait Anxiety Inventory to assess trait anxiety; SDI = Sexual Desire Inventory to assess dyadic (i.e. with partner) and solitary sexual desire; SQOL = Sexual Quality of Life score; SwLS = Satisfaction with Life Scale to assess satisfaction with life as a whole; SHS = Subjective Happiness Scale. Results confirmed no active depression, anxiety trait or underlying erectile disorder that could affect fMRI analysis. Baseline scent ratings scored on a scale of 0 to 5. Pleasantness: 0 = very unpleasant, 5 = very pleasant. Feminine: 0 = masculine scent, 5 = feminine scent. Data presented as mean±SEM.

Supplemental Table S2: Baseline clinical and psychometric characteristics on kisspeptin and placebo visits.

		Kisspeptin Visit (*n* = 33)	Placebo Visit (*n* = 33)
		Mean ± SEM	Mean ± SEM
**Baseline Reproductive Hormones**			
	Kisspeptin (pmol/L)	21.1 ± 4.0	14.7 ± 1.5
	LH (IU/L)	2.9 ± 0.2	3.0 ± 0.2
	FSH (IU/L)	3.0 ± 0.3	2.9 ± 0.3
	Testosterone (nmol/L)	21.3 ± 1.1	20.2 ± 1.1
	Cortisol (nmol/L)	295.2 ± 16.1	309.8 ± 15.2
**Baseline SADI scores**			
	Evaluative	27.2 ± 4.0	28.9 ± 3.8
	Negative	16.2 ± 2.8	18.1 ± 2.8
	Physiological	15.5 ± 2.7	16.7 ± 2.6
	Motivational	14.5 ± 2.6	15.7 ± 2.3
**Baseline POMS2A scores**			
	Total Mood Disturbance	45.4 ± 1.3	45.2 ± 1.4
	Anger-Hostility	40.4 ± 0.9	41.0 ± 1.0
	Confusion-Bewilderment	42.4 ± 1.2	42.2 ± 0.9
	Depression-Dejection	43.9 ± 1.0	43.6 ± 0.8
	Fatigue-Inertia	41.5 ± 1.3	41.9 ± 1.5
	Tension-Anxiety	43.5 ± 1.4	44.4 ± 1.7
	Vigor-Activity	42.7 ± 1.5	45.7 ± 1.9
	Friendliness	47.4 ± 1.8	50.7 ± 1.6

LH = Luteinizing Hormone, FSH = Follicle Stimulating Hormone, SADI = Sexual Arousal and Desire Inventory, POMS2A = Profile of Mood States 2 for Adults. *n*=17 participants received placebo and *n*=16 received kisspeptin at first visit. No significant difference in parameters between visits assessed by multi-level linear regression, adjusted for visit order. Hormone levels were within the normal expected physiological ranges. Data presented as mean±SEM.

Supplemental Table S3: Change in psychometric scores during kisspeptin and placebo visits.

		**Kisspeptin Visit (*n* = 33)**	**Placebo Visit (*n* = 33)**
		Mean ± SEM	Mean ± SEM
**Change in SADI scores**			
	Evaluative	0.0 ± 2.9	3.0 ± 3.2
	Negative	0.8 ± 1.4	-1.8 ± 1.8
	Physiological	1.7 ± 1.4	3.3 ± 2.0
	Motivational	-0.3 ± 1.4	-0.1 ± 1.7
**Change in POMS2A scores**			
	Total Mood Disturbance	1.4 ± 1.3	-0.6 ± 1.0
	Anger-Hostility	0.7 ± 0.7	-0.5 ± 0.4
	Confusion-Bewilderment	1.3 ± 1.3	0.5 ± 0.8
	Depression-Dejection	-1.1 ± 0.5	-0.8 ± 0.6
	Fatigue-Inertia	3.4 ± 1.9	1.6 ± 1.3
	Tension-Anxiety	-3.5 ± 1.0	-5.6 ± 1.1
	Vigor-Activity	-3.7 ± 1.8	-2.1 ± 2.0
	Friendliness	-4.1 ± 1.7	-3.5 ± 1.1

SADI = Sexual Arousal and Desire Inventory, POMS2A = Profile of Mood States 2 for Adults. *n*=17 participants received placebo and *n*=16 received kisspeptin at first visit. No significant difference in parameters between visits assessed by multi-level linear regression, adjusted for visit order. Data presented as mean±SEM.

Supplemental Table S4: Structures with enhanced activation by kisspeptin on whole brain analysis.

Contrast	Region	Cluster Peak (MNI152 Space)					
		Right			Left		
Kisspeptin > Placebo		x	y	z	x	y	z
**Olfactory Task**							
Scent	Putamen	32	-16	4	-32	-6	4
	Globus Pallidus	24	-20	38	-24	-8	4
	Insula	34	-22	6	-40	-16	-2
	Caudate				-8	6	10
	Amygdala	20	-8	-14			
	Thalamus	6	-16	2	-8	-12	6
	Posterior Cingulate Cortex (PCC)	26	-58	6	-10	-70	14
	Superior Temporal Gyrus	66	-20	0	-64	-28	0
	Middle Temporal Gyrus	60	-30	-8	-50	-30	-8
	Heschl's Gyrus	44	-22	8	-40	-28	10
**Facial Attractiveness Task**							
Low Attractiveness	Superior Frontal Gyrus				-28	20	46
Medium Attractiveness	Medial Prefrontal Cortex	6	56	6	-10	54	8
	Superior Frontal Gyrus	-22	32	46			
	Anterior Cingulate Gyrus	4	24	18	-4	24	18
High Attractiveness	Medial Prefrontal Cortex	6	56	8	-12	54	8
	Superior Frontal Gyrus				-22	30	46

Data derived from whole brain analysis during the olfactory task for the scent trials and the facial attractiveness tasks for low, medium and high attractiveness images (*n*=33). Co-ordinates represented by x, y, z are derived from the MNI152 stereotactic coordinate space. Coordinates represent the center of discrete activation clusters observed in the group-level analyses of treatment effects (kisspeptin vs. placebo), with statistical maps thresholded at $Z = 2.3$, $P < 0.05$ (cluster-corrected for multiple comparisons).
